# Supplementary material for: Control of the polyamine biosynthesis pathway by G2-quadruplexes
Source: eLife. 2018 Jul 31;7:e36362. doi: 10.7554/eLife.36362 (PMC6067879; doi:10.7554/eLife.36362)
Supplement: Figure 2—source data 1. — Assessment of PQS conservation using H-QGRS (Menendez et al., 2012), with the exception of SMS3Q1 and ARG23Q1 whose stability score is below that set by H-QGRS. SMS3Q1 and ARG23Q1 conservation was assessed through manual gene alignment. Conservation is dictated by factors such as composition, location and predicted stability. Human-mouse conservation was not observed for the reference NRAS, nor for ARG25Q1, ARG23Q1, OAZ25Q1, OAZ25Q2, SMS3Q1, OAZ13Q1 and OAZ33Q1, for which Human-primate conservation was assessed. For many PQS's, primate sequences are either not available or the UTRs are not defined (OAZ13Q1). Human-primate conservation was not observed for ARG25Q1, OAZ25Q1 and OAZ25Q2. Human-primate conservation has been reported for NRAS (Kumari et al., 2007). [file elife-36362-fig2-data1.pptx]

## Slide 1
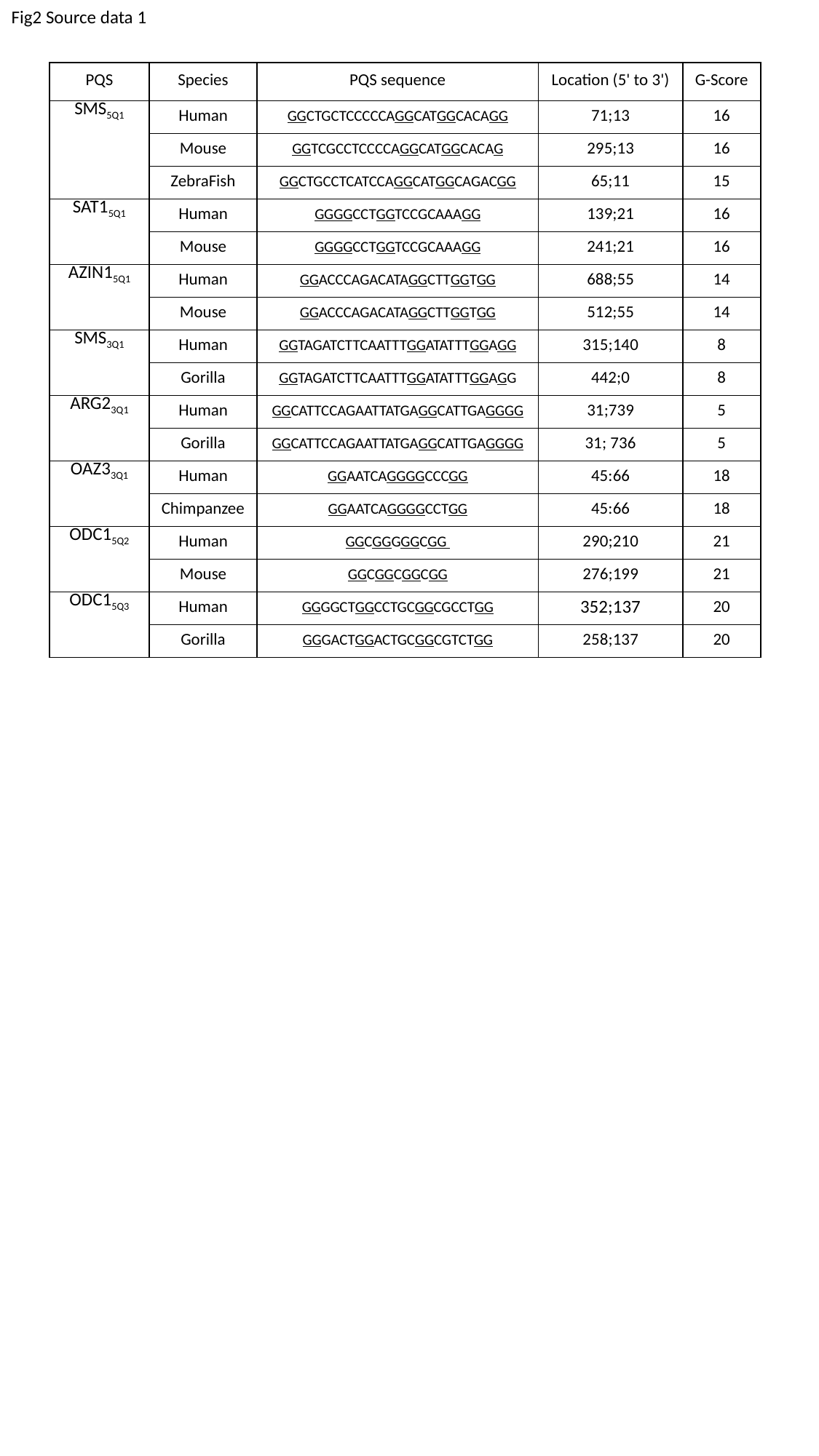

Fig2 Source data 1
| PQS | Species | PQS sequence | Location (5' to 3') | G-Score |
| --- | --- | --- | --- | --- |
| SMS5Q1 | Human | GGCTGCTCCCCCAGGCATGGCACAGG | 71;13 | 16 |
| | Mouse | GGTCGCCTCCCCAGGCATGGCACAG | 295;13 | 16 |
| | ZebraFish | GGCTGCCTCATCCAGGCATGGCAGACGG | 65;11 | 15 |
| SAT15Q1 | Human | GGGGCCTGGTCCGCAAAGG | 139;21 | 16 |
| | Mouse | GGGGCCTGGTCCGCAAAGG | 241;21 | 16 |
| AZIN15Q1 | Human | GGACCCAGACATAGGCTTGGTGG | 688;55 | 14 |
| | Mouse | GGACCCAGACATAGGCTTGGTGG | 512;55 | 14 |
| SMS3Q1 | Human | GGTAGATCTTCAATTTGGATATTTGGAGG | 315;140 | 8 |
| | Gorilla | GGTAGATCTTCAATTTGGATATTTGGAGG | 442;0 | 8 |
| ARG23Q1 | Human | GGCATTCCAGAATTATGAGGCATTGAGGGG | 31;739 | 5 |
| | Gorilla | GGCATTCCAGAATTATGAGGCATTGAGGGG | 31; 736 | 5 |
| OAZ33Q1 | Human | GGAATCAGGGGCCCGG | 45:66 | 18 |
| | Chimpanzee | GGAATCAGGGGCCTGG | 45:66 | 18 |
| ODC15Q2 | Human | GGCGGGGGCGG | 290;210 | 21 |
| | Mouse | GGCGGCGGCGG | 276;199 | 21 |
| ODC15Q3 | Human | GGGGCTGGCCTGCGGCGCCTGG | 352;137 | 20 |
| | Gorilla | GGGACTGGACTGCGGCGTCTGG | 258;137 | 20 |
