# Supplementary material for: Control of the polyamine biosynthesis pathway by G2-quadruplexes
Source: eLife. 2018 Jul 31;7:e36362. doi: 10.7554/eLife.36362 (PMC6067879; doi:10.7554/eLife.36362)
Supplement: Supplementary file 1. [file elife-36362-supp1.docx]

# **Supplementary File 1**

**Control of the polyamine biosynthesis pathway by G_2_-quadruplexes**

Helen L. Lightfoot^1^, Timo Hagen^1^, Antoine Cléry^2^, Frédéric H.-T. Allain^2^ and Jonathan Hall^1^*

^1^Institute of Pharmaceutical Sciences, Department of Chemistry and Applied Biosciences; ^2^Institute of Molecular Biology and Biophysics, Department of Biology, ETH Zürich, CH-8093 Zürich

* To whom correspondence should be addressed. Tel: +41 44 633 74 35; Email: [jonathan.hall@pharma.ethz.ch](mailto:jonathan.hall@pharma.ethz.ch)

Contents

[Supplementary File 1 1](#_Toc515801155)

[Table S1: PQS's and PQS mutations in the UTRs of the PSPs 1](#_Toc515801156)

[Table S2: Reported functional motifs in the UTRs containing G_2_-PQS's 2](#_Toc515801157)

[Table S3: PSP genes and transcripts 4](#_Toc515801158)

[Table S4: Reporter insert primers for UTR amplification (UTRs >150 nt) 5](#_Toc515801159)

[Table S5: Oligonucleotide reporter-insert templates and primers for UTRs ≤150 nt 6](#_Toc515801160)

[Table S6: Primers sequence (5' to 3') used for quadruplex mutagenesis 7](#_Toc515801161)

[Table S7 (accompanying Excel file): Sequences of plasmids 8](#_Toc515801162)

## Table S1: PQS's and PQS mutations in the UTRs of the PSPs

| **PQS name** | **PQS^a^** | **PQS mutation ^b^** | **UTR^c^** |
| --- | --- | --- | --- |
| ARG2_5Q1_ | GGCUGGUUGGAGGCGCGGGCGGCGGG | AACUAAUUGGAGGCGCAAACAACAAA | 180 |
| ARG2_5Q2_ | GGCGGGCGGACGCUGGCGCGGG | AACGAACAAACGCUGGCGCGGG |  |
| ARG2_5Q3_ | GGCGGGCGGUGG | GGCGGGCAAUAA |  |
| AMD1_5Q1_ | GGCGGCGGCAGCGGCGGG | AACAACAACAGCGGCGGG | 150* |
| AZIN1_5Q1_ | GGACCCAGACAUAGGCUUGGUGG | GGACCCAGACAUAGGCUUAAUAA | 574* |
| OAZ1_5Q1_ | GGCGGCGGCGCGG | AACAACGGCGCGG | 78 |
| OAZ1_5Q2_ | GGUUUUCCUGGUUUCGGACCCCAGCGG | GGUUUUCCUAAUUUCAAACCCCAGCGG |  |
| OAZ2_5Q1_ | GGCACUCGGGGGCGGGGCGGCGG | GGCACUCAAGAAd_9_GG | 233 |
| OAZ2_5Q2_ | GGCGGUGGCGGCCGGGGAGGGUCAGUUGG | GGCGGUGGCAACCGAAGAAAGUCAGUUGG |  |
| OAZ2_5Q3_ | GGCAGGCGCUCGCUGAGGCAAAAGG | AACAAACGCUCGCUGAGGCAAAAGG |  |
| OAZ2_5Q4_ | GGCGCUCGGCCCGCGGCCUGACAGG | AACGCUCAACCCGCGGCCUGACAGG |  |
| OAZ3_5Q1_ | GGUGCAGGCGCGGCUUCCGG | GGUGCAAACGCAACUUCCGG | 167 |
| ODC_5Q1_ | GGCGGGCGGGAGCCAGGCGCUGACGG | GACGAGCGAGAGCCAAGCGCUGACGG | 301* |
| ODC_5Q2_ | GGCGGGGGCGG | AACAAGAACGG |  |
| ODC_5Q3_ | GGGGCUGGCCUGCGGCGCCUGG | AAGACUAACCUGCGGCGCCUGG |  |
| ODC_5Q4_ | GGCACACGCAGAGGGAUUUGGAAUUCCUGG | GGCACACGCAGAGAGAUUUAGAAUUCCUGG |  |
| SAT1_5Q1_ | GGGGCCUGGUCCGCAAAGG | AAGACCUGGUCCGCAAAGG | 179 |
| SMOX_5Q1_ | GGCUCAGCAGGGAAAGGUUCCUAGAAGG | AACUCAGCAGAGAAAGGUUCCUAGAAGG | 69 |
| SMS_5Q1_ | GGCUGCUCCCCCAGGCAUGGCACAGG | GGCUGCUCCCCCAAACAUAACACAGG | 110 |
| SRM_5Q1_ | GGCGGAGCUGGUCCCGUUGUGCUGCGG | AACAAAGCUGGUCCCGUUGUGCUGCGG | 81 |
| PAOX_3Q1_ | GGGGGUAGGCUGG | GAGAGUAGACUGA | 315 |
| PAOX_3Q2_ | GGUCCUCUGGUUUUUGGUAACCAGG | AGUCCUCUGAUUUUUGGUAACCAGA |  |
| ARG2_3Q1_ | GGCAUUCCAGAAUUAUGAGGCAUUGAGGGG | GGCAUUCCAGAAUUAUGAAGCAUUGAGAAG | 800 |
| ARG2_3Q2_ | GGUUAGACCUGGGACCACGGCUGG | AGUUAGACCUGAGACCACGACUGA |  |
| AMD1_3Q1_ | GGGGAAUGGGGGAGAUGGUCCCUGGG | GGGGAAUGAGAGAGAUGAUCCCUGAG | 2097 |
| AZIN1_3Q1_ | GGCUAAAACUUUGGGAAAGGGG | GGCUAAAACUUUGAGAAAGAAG | 744 |
| OAZ1_3Q1_ | GGGGCUGGGCAUCCGGCCCCUGG | GAAGCUGAGCAUCCGACCCCUGG | 382 |
| OAZ1_3Q2_ | GGCCACCCCUUGUCAGCCGGGUGGGUAGG | AGCCACCCCUUGUCAGCCGAGUGAGUAGA |  |
| OAZ3_3Q1_ | GGAAUCAGGGGCCCGG | GGAAUCAGAGGCCCGA | 110 |
| ODC_3Q1_ | GGUGGUGGGACCUACUUAAGUCUGACGG | GGUGAUGGGACCUACUUAAGUCUGACAG | 756 |
| ODC_3Q2_ | GGGAAGGUGUGGGAGG | GAGAAGGUGUGGGAGG |  |
| SMOX_3Q1_ | GGCUGGGCCGUGAGCAGGUGGG | GGCUGAGCCGUGAGCAGGUGAG | 340 |
| SMS_3Q1_ | GGUAGAUCUUCAAUUUGGAUAUUUGGAGG | GGUAGAUCUUCAAUUUGAAUAUUUGAAAG | 484 |
| SRM_3Q1_ | GGACCUCGGACCUUGGAGCCUGCGG | GGACCUCAGACCUUGAAGCCUGCGA | 272 |
| SRM_3Q2_ | GGUGCCUCGGCCCCUCCAGCCCCGGGCCGG | GAUGCCUCAGCCCCUCCAGCCCCGAGCCGG |  |

**Table S1**: PQSs and the respective PQS mutations (QMs) in the UTRs of the PSPs. **a**. PQS motifs are represented as the gene name with the UTR (3' or 5') and PQS number (Q_n_; where n=≥1). Underlined guanines are those forming the most stable predicted PQS from all possible PQS’s predicted by QGRS Mapper. **b**. Selected G`s are mutated. **c**. *portion of the UTR used in this study (AMD1: 150-AUG; AZIN1: 720-241 and 95-AUG; ODC1: 301-AUG) (related to **Fig. 1**).

## Table S2: Reported functional motifs in the UTRs containing G_2_-PQS's

| PQS | PQS location (5' to 3')  (UTR) | | Known functional  elements (UTR) | | Overlap  with PQS |
| --- | --- | --- | --- | --- | --- |
|  | **Natural** | **Construct** | **Natural** | **Construct** |  |
| ARG2_5Q1_ | 19; 180 | 19; 180 | - | - | - |
| OAZ1_3Q1_ | 9; 350 | 9; 350 | - | - | - |
| ODC1_5Q2_ | 80; 210 | 80; 210 | IRES (483; 16)([1](#_ENREF_1)) | IRES (273; 16) | - |
| ODC1_5Q3_ | 432; 137 | 142; 137 |  |  |  |
| OAZ2_5Q1_ | 10; 200 | 10; 200 | - | - | - |
| OAZ2_5Q2_ | 43; 161 | 43; 161 | - | - | - |
| SAT1_5Q1_ | 139; 21 | 139; 21 | uORF (partial)([2](#_ENREF_2))  ( 74 AUG) | uORF (partial)  ( 74 AUG) | PQS 53 nts downstream of  uORF AUG |
| AZIN1_5Q1_ | 688; 55 | 496; 55 | uORF([3](#_ENREF_3))  (335; 277) | uORF  (289; 131) | - |
| SMS_5Q1_ | 71; 13 | 71; 13 | - | - | - |
| ARG2_3Q1_ | 31; 739 | 31; 739 | - | - | - |
| SMS_3Q1_ | 315; 140 | 315; 140 | - | - | - |
| OAZ3_3Q1_ | 45; 49 | 45; 49 | - | - | - |

**Table S2** (related to **Fig. 1c**): Reported functional motifs in the UTRs containing functional PQSs. X;Z were X represents the distance of the PQS motif from the 5' end of the UTR and Z represents the distance of the PQS motif from the 3' end of the UTR. Natural: natural UTR. Construct: UTR downstream (3' UTR) or upstream (5' UTR) of the *Renilla* reporter gene used. IRES: internal ribosome entry site. uORF: Upstream Open Reading Frame. The SAT1 uORF continues into the main ORF, which is not present in the reporter construct. ODC1_5Q2_/ODC1_5Q3_ and AZIN1_5Q1_ are not positioned within or adjacent to the IRES and uORF, respectively. SAT1_5Q1_ is positioned 53 nt downstream of the uORF AUG, however this uORF is incomplete. See also **Supplementary Table S7**.

## Table S3: PSP genes and transcripts

| Gene | Transcript ID | CCDS | Ref Seq |
| --- | --- | --- | --- |
| ARG1 ENSG00000118520 | ENST00000356962 | CCDS59038 | NM_001244438 NP_001231367 |
| PAOX ENSG00000148832 | ENST00000278060 | CCDS7683 | NM_152911 NP_690875 |
| ARG2  ENSG00000081181 | ENST00000261783 | CCDS9785 | NM_001172  NP_001163 |
| AMD1 ENSG00000123505 | ENST00000368885 | CCDS5086 | NM_001287214 NM_001634 NP_001274143 NP_001625 |
| AZIN1 ENSG00000155096 | ENST00000347770 | CCDS6295 | NM_015878 NP_056962 |
| OAZ1 ENSG00000104904 | ENST00000602676 | CCDS58639 | NM_004152 NP_004143 |
| OAZ2 ENSG00000180304 | ENST00000326005 | CCDS58372 | NM_001301302 NM_002537 NP_001288231 NP_002528 |
| OAZ3 ENSG00000143450 | ENST00000321531 | CCDS58028 | NM_001134939 NP_001128411 |
| ODC1 ENSG00000115758 | ENST00000234111 | CCDS1672 | NM_001287188 NM_002539 NP_001274117 NP_002530 |
| SAT1 ENSG00000130066 | ENST00000379270 | CCDS14207 | NM_002970 NP_002961 |
| SMOX ENSG00000088826 | ENST00000346595.6 | CCDS13077 | NM_175841  NP_787035 |
| SMS ENSG00000102172 | ENST00000404933  ENST00000379404 | CCDS14203  CCDS59161 | NM_004595 NP_004586  NM_001258423  NP_001245352 |
| SRM ENSG00000116649 | ENST00000376957 | CCDS125 | NM_003132 NP_003123 |

**Table S3**: PSP genes and transcripts considered for this study. All transcripts were designated as merged Ensembl and HAVANA.

## Table S4: Reporter insert primers for UTR amplification (UTRs >150 nt)

| **Insert UTR** | **Nested primers**  **(if applicable)** | **Exact primer** |
| --- | --- | --- |
|  |  |  |
| ARG2 5’-UTR | F: GCGAAGAAGGTGTGCCGGGG  R: ACAGCCACGGAGTGGACGGA | F: **RS-**GCGAAGAAGGTGTGCCGGGG  R: **RS-**AACATGATCCGCAGCACTGAGAATCTCC |
| AZIN1 (-720;-241\|-95;0) 5’-UTR | cDNA plasmid template | F: **RS-**ATCGCGGCCGCAGTTTTTCCTT  R: **RS-**AACATCTCAGCCGTATTCCACAAAG |
| OAZ2 5’-UTR | F: ATGCAGATGAGGCACTCGGGGG  R: TGAGGGGCATCGGAGCACCA | F: **RS-**ATGCAGATGAGGCACTCGGGGG  R: **RS-**AACATCCTCGGCGGCTCTGCTCA |
| ODC1 (-301;0) 5’-UTR | cDNA plasmid template | F: **RS-**CCGCCGCCCCTCTGCCAG  R: **RS-**AACATGATTTCTTGATGTTCCTATGGAAAACTAAGAGA |
| SAT1 5’-UTR | F: GCGCAGCTCTTAGTCGCGG  R: TCGGCACTTCTGCAACCAGGC | F: **RS-**GCGCAGCTCTTAGTCGCGG  R: **RS-**AACATTTTCGTCTTTTGCTTTTCTTC |
| PAOX 3’-UTR | F: CCCCATATGTGATCCAGGAG  R: GTTGTGGGGATTGGAGACAC | F: **RS2-**CAGCTCCAGATCCTGTTTGC  R: **RS3-**CTCAGCTCAAGTCAACCCTGG |
| ARG2 3’-UTR | F: CCAGTGGGATGACCCTCA  R: ATGAATGTGGGGTGTGTGTG | F: **RS2-**TGCTTCAAGCTTTGGTCAGA  R: **RS3-**AAAGAACCTTATGAGTACTTCCTGCT |
| AZIN1 3’-UTR | F: CTGCGATGTAACGAGGTGATAGG  R: GGCTTCTCTAACCCAGTATTTCC | F: **RS2-**ACAGGCATTAACGCTTCTTTAGAT  R: **RS3-**CACAATGATTTACCAAACACTTTACT |
| AMD1 3’ UTR | F: CCAAACTGATGGGTTCCTCCTG  R: GCCATCACACTTGGCTTTGTC | F: **RS2-**AAGCAGCAACAACAGCAGAG  R: **RS3-**TTTCTGAATGTCACTAGACAAT |
| OAZ1 3’-UTR | F: TCTCCTGGAGTTCGCTGAG  R: GCACAGACATCCCCATGC | F: **RS2-**CGGGAGAGGAGGAGGAGTAG  R: **RS3-**TCGGGTGTAATCACTTTATTGG |
| OAZ2 3’-UTR | F: GGTCTAGGAAGGTGGTGATGG  R: GGAATGGGAATGTCAGGAGCT | F: **RS2-**CAGCTCCACTCCTGAAGACC  R: **RS3-**GGTAGGGAGTTGGTCCTAGTT |
| ODC1 3’-UTR | F: CATGCAGCAATTCCAGAACC  R: GAGAGCCTGCCACACTTTG | F: **RS2-**TGTAGATAGCACTCTGGTAGCTGT  R: **RS3-**TAGGGACTTGCTGTTGCTGAA |
| SMOX 3’-UTR | F: AAAGAGCCAAGGGAAGAAGC  R: TTCACTCCAGGCAGTGTGTC | F: **RS2-**GCCTCATTGAGATGTACCGAGAC  R: **RS3-**TGAGAAAACCAGAGAGAAGCTGAG |
| SMS 3’-UTR | F: CTCGAGATTCCTTGACTCCCTGTCCA  R: GCGGCCGCTGGGCTGAAATAAAACGACA | F: **RS2-**AGATCAGTAGCCCCTAATCACA  R: **RS3-**TCTCTTTTGTAATTTTAATTGAAGCAA |
| SRM 3’-UTR | F: CTCGAGCTCCTGACTGGCCTCATGTC  R: GCGGCCGCCAGCCAAGGTCAGGAGACAC | F: **RS2-**CCCTGAATGATGTGAGCTGAG  R: **RS3-**CTATAAATACACGTGTTTGGTGAGTGA |

**Table S4**: Reporter insert primers (sequence 5' to 3') for UTR amplification. RS: Restriction Site. **RS**: AAAGCTAGC **RS2**: AAACTCGAG **RS3**: AAAGCGGCCGC. Underlined: *Renilla* start codon.

## Table S5: Oligonucleotide reporter-insert templates and primers for UTRs ≤150 nt

| **Insert UTR** | **DNA oligonucleotide templates** | **Amplification primers** |
| --- | --- | --- |
| AMD1 5’-UTR | AGTTAATATAAAATTATAGCAAAAAAAAAAAGGAACCTGAACTTTAGTAACACAGCTGGAACAATCCGCAGCGGCGGCGGCAGCGGCGGGAGAAGAGGTTTAATTTAGTTGATTTTCTGTGGTTGTTGGTTGTTCGCTAGTCTCACGGTG | F: **RS-**AGTTAATATAAAATTATAGCAAAAAAA  R: **RS-**AACATCACCGTGAGACTAGCGAA |
| OAZ1 5’-UTR | P-**RS** -TTTTGCGAACGGCGAGCAGCGGCGGCGGCGCGGAGAGACGCAGCGGAGGTTTTCCTGGTTTCGGACCCCAGCGGCCGGATGTT -**RS**- P` | P and P` rev com |
| SMS 5’-UTR | P-**RS** -GGCCTCCCCGGGCGCAGCACACTCCCAGCCGGCCGCAGCCTGACACGCCGCGCGGCCCCCCAGTCTCCCGCGGCTGCTCCCCCAGGCATGGCACAGGGCCTCGCCTCACTATGTT - **RS**- P` | P and P` rev com |
| SRM 5’-UTR | P-**RS** -GGCGGAGCTGGTCCCGTTGTGCTGCGGCGCCGCGCGGCCTGCAGTCCCGGGCCCGCGCCCCGCGCCGCCCGCCCGCCCGCCATGTT -**RS**-P` | P and P` rev com |
| OAZ3 5’-UTR | P-**RS** -GCGCCTCGGCCCGGGCCTTACTCTCCACCGACTGCGTCAGGATGAGCTCCAGGTTTTCTTTGGGCCGATGCTTCGTGTCCTCCACCAGCTTATAGACGCGATGTCGCCGGTGCAGGCGCGGCTTCCGGCCCTCCCCGGCCAGCGGTACCTTCCACCAGCGCTCCACGATGTT **RS** - P` | P and P` rev com |
| OAZ3QM 5’-UTR | P-**RS** -GCGCCTCGGCCCGGGCCTTACTCTCCACCGACTGCGTCAGGATGAGCTCCAGGTTTTCTTTGGGCCGATGCTTCGTGTCCTCCACCAGCTTATAGACGCGATGTCGCCGGTGCAAACGCAACTTCCGGCCCTCCCCGGCCAGCGGTACCTTCCACCAGCGCTCCACGATGTT **RS** - P` | P and P` rev com |
| SMOX 5’-UTR | P-**RS** -CGCCGCTCGCCGCAGACTTACTTCCCCGGCTCAGCAGGGAAAGGTTCCTAGAAGGTGAGCGCGGACGGT **RS** - P` | P and P` rev com |
| OAZ3 3’-UTR | P-**RS2**-ACATGCTTATTCCAACGCTTTGAGGGGCTGGAAGCCTTGACACATGGAATCAGGGGCCCGGGATGTGATTCAGGACACTTTCCATCCTAGGAATAAAGGGTAGTGCAATCATC-**RS3**-P` | P and P` rev com |

P:CCTCCACTTCAGCCAGGA P`: GCGAGGTCCGAAGACTCA

**Table S5**: Oligonucleotide reporter-insert templates and amplification primers (sequence 5' to 3') for UTRs equal to and less than 150 nt. **RS**: AAAGCTAGC. **RS2**: CTCGAG. **RS3**: GCGGCCGC. Underlined: *Renilla* start codon. rev com: reverse complement.

## Table S6: Primers sequence (5' to 3') used for quadruplex mutagenesis

## Table S7 (accompanying Excel file): Sequences of plasmids

1. Sammons, M.A., Antons, A.K., Bendjennat, M., Udd, B., Krahe, R. and Link, A.J. (2010) ZNF9 activation of IRES-mediated translation of the human ODC mRNA is decreased in myotonic dystrophy type 2. *PloS one*, **5**, e9301.

2. Perez-Leal, O. and Merali, S. (2012) Regulation of polyamine metabolism by translational control. *Amino acids*, **42**, 611-617.

3. Ivanov, I.P., Loughran, G. and Atkins, J.F. (2008) uORFs with unusual translational start codons autoregulate expression of eukaryotic ornithine decarboxylase homologs. *Proceedings of the National Academy of Sciences of the United States of America*, **105**, 10079-10084.
